# Treatment strategy for insomnia disorder: Japanese expert consensus

**DOI:** 10.3389/fpsyt.2023.1168100

**Published:** 2023-05-09

**Authors:** Yoshikazu Takaesu, Hitoshi Sakurai, Yumi Aoki, Masahiro Takeshima, Kenya Ie, Kentaro Matsui, Tomohiro Utsumi, Akiyoshi Shimura, Isa Okajima, Nozomu Kotorii, Hidehisa Yamashita, Masahiro Suzuki, Kenichi Kuriyama, Eiji Shimizu, Kazuo Mishima, Koichiro Watanabe, Ken Inada

**Affiliations:** ^1^Department of Neuropsychiatry, Graduate School of Medicine, University of the Ryukyus, Okinawa, Japan; ^2^Department of Neuropsychiatry, Faculty of Medicine, Kyorin University, Tokyo, Japan; ^3^Psychiatric and Mental Health Nursing, St. Luke’s International University, Tokyo, Japan; ^4^Department of Neuropsychiatry, Akita University Graduate School of Medicine, Akita, Japan; ^5^Division of General Internal Medicine, Department of Internal Medicine, St. Marianna University School of Medicine, Kawasaki, Japan; ^6^Division of General Internal Medicine, Department of Internal Medicine, Kawasaki Municipal Tama Hospital, Kawasaki, Japan; ^7^Department of Clinical Laboratory, National Center Hospital, National Center of Neurology and Psychiatry, Tokyo, Japan; ^8^Department of Sleep-Wake Disorders, National Institute of Mental Health, National Center of Neurology and Psychiatry, Tokyo, Japan; ^9^Department of Psychiatry, The Jikei University School of Medicine, Tokyo, Japan; ^10^Department of Psychiatry, Tokyo Medical University, Tokyo, Japan; ^11^Department of Psychological Counseling, Faculty of Humanities, Tokyo Kasei University, Tokyo, Japan; ^12^Kotorii Isahaya Hospital, Nagasaki, Japan; ^13^Department of Psychiatry, School of Medicine, Kurume University, Fukuoka, Japan; ^14^Minna No Sleep and Stress Care Clinic, Hiroshima, Japan; ^15^Department of Psychiatry, Nihon University School of Medicine, Tokyo, Japan; ^16^Department of Psychiatry, National Center Hospital, National Center of Neurology and Psychiatry, Tokyo, Japan; ^17^Research Center for Child Mental Development, Chiba University, Chiba, Japan; ^18^Department of Cognitive Behavioral Physiology, Graduate School of Medicine, Chiba University, Chiba, Japan; ^19^Department of Psychiatry, School of Medicine, Kitasato University, Tokyo, Japan

**Keywords:** insomnia, hypnotics, cognitive behavioral therapy, sleep hygiene education (SHE), orexin receptor antagonists, benzodiazepine

## Abstract

**Purpose:**

There is a lack of evidence regarding answers for clinical questions about treating insomnia disorder. This study aimed to answer the following clinical questions: (1) how to use each hypnotic and non-pharmacological treatment differently depending on clinical situations and (2) how to reduce or stop benzodiazepine hypnotics using alternative pharmacological and non-pharmacological treatments.

**Methods:**

Experts were asked to evaluate treatment choices based on 10 clinical questions about insomnia disorder using a nine-point Likert scale (1 = “disagree” to 9 = “agree”). The responses of 196 experts were collected, and the answers were categorized into first-, second-, and third-line recommendations.

**Results:**

The primary pharmacological treatment, lemborexant (7.3 ± 2.0), was categorized as a first-line recommendation for sleep initiation insomnia, and lemborexant (7.3 ± 1.8) and suvorexant (6.8 ± 1.8) were categorized as the first-line recommendations for sleep maintenance insomnia. Regarding non-pharmacological treatments for primary treatment, sleep hygiene education was categorized as the first-line recommendation for both sleep initiation (8.4 ± 1.1) and maintenance insomnia (8.1 ± 1.5), while multicomponent cognitive behavioral therapy for insomnia was categorized as the second-line treatment for both sleep initiation (5.6 ± 2.3) and maintenance insomnia (5.7 ± 2.4). When reducing or discontinuing benzodiazepine hypnotics by switching to other medications, lemborexant (7.5 ± 1.8) and suvorexant (6.9 ± 1.9) were categorized as first-line recommendations.

**Conclusion:**

Expert consensus indicates that orexin receptor antagonists and sleep hygiene education are recommended as first-line treatments in most clinical situations to treat insomnia disorder.

## 1. Introduction

Insomnia is a common disorder, with an incidence of 15–20% (1) in the general population. Due to the prevalence of insomnia, not only sleep disorder specialists but also primary care physicians usually treat patients with insomnia disorder in clinical settings. Thus, treatment guidelines are necessary for clinicians to manage patients with insomnia disorder. Several clinical guidelines for insomnia disorder based on scientific evidence have been published worldwide (2–4). Although these guidelines provide credible and robust evidence for treating insomnia disorder, it is difficult to interpret these them in clinical settings because they usually provide only general recommendations. Practical guidelines providing information on clinical questions, which are patient-dependent, and clinical situations beyond established evidence are warranted for primary care physicians to manage and develop an effective treatment strategy for insomnia disorder.

Benzodiazepines and benzodiazepine receptor agonists (BZDs) are commonly used to treat insomnia disorder. However, previous studies have reported several disadvantages to high-dose and long-term use of BZD hypnotics, including cognitive dysfunction (5), risk of falls and fractures (6), and development of tolerance and dependence (7, 8). Thus, BZDs should preferably be used only for short-term treatment (up to 4 weeks) to prevent the disadvantages associated with its long-term use (4). However, despite these warnings regarding the risks associated with high-dose, long-term BZD use, BZD hypnotics are commonly prescribed worldwide to patients with insomnia disorder (9, 10). Thus, a treatment strategy to avoid the use of BZD hypnotics, implement alternative treatment, and discontinuation BZD is necessary for the proper management of patients with insomnia disorder in clinical settings.

Orexin receptor antagonists and melatonin receptor agonists are alternative treatments for insomnia disorder. Two types of orexin receptor antagonists—suvorexant and lemborexant—are available in Japan. Both suvorexant (11) and lemborexant (12) are reportedly effective and safe, and do not cause cognitive dysfunction, risk of falling, and development of dependence within at least 1 year. Ramelteon, a melatonin receptor agonist, is also a candidate for alternative hypnotic treatment that has a good safety profile (13). Trazodone and quetiapine are commonly prescribed for insomnia disorder in clinical settings, although the risk/benefit ratio is still unclear (2). Kampo is also frequently prescribed for insomnia treatment in Japan, regardless of the lack of clear evidence (14). However, clinicians are still uncertain about how to use each medication differently since they are situation-dependent and because the clinical guidelines do not include a clear strategy.

Non-pharmacological treatments, such as sleep hygiene education and cognitive behavioral therapy for insomnia (CBT-I), are also useful to manage insomnia disorder. Because there is substantial evidence on the effectiveness of CBT-I in patients with insomnia disorder, most clinical guidelines recommend CBT-I as first-line treatment (3, 4, 15). However, because structured and multicomponent CBT-I needs a well-trained therapist, it is difficult to provide it for all patients in Japan and many other countries due to a lack of resources. Hence, a more realistic non-pharmacological treatment strategy should be considered, especially in primary care settings.

Accordingly, we developed an expert consensus focusing on (1) how to use each hypnotic and non-pharmacological treatment differently depending on individual clinical situations and (2) how to reduce or discontinue BZD hypnotics by alternative pharmacological and non-pharmacological treatments beyond the current scientific evidence.

## 2. Materials and methods

### 2.1. Study design

The task force to answer the two aforementioned questions comprised 14 insomnia disorder specialists, one primary care provider, one expert psychologist, and one expert nurse (all authors). The task force identified 10 clinical questions on the management of insomnia disorder without psychiatric comorbidity based on the Diagnostic and Statistical Manual (DSM-5) (16) to develop a treatment strategy for insomnia disorder. The questions were as follows: Q1, Which pharmacological treatments would you recommend for sleep initiation insomnia in primary treatment?; Q2, Which pharmacological treatments would you recommend for sleep maintenance insomnia in primary treatment?; Q3, Which non-pharmacological treatments would you recommend for sleep initiating insomnia in primary treatment?; Q4, Which non-pharmacological treatments would you recommend for sleep maintenance insomnia in primary treatment?; Q5, Which pharmacological treatments would you recommend if a BZD does not improve insomnia symptoms?; Q6, Which non-pharmacological treatments would you recommend if BZDs do not improve insomnia symptoms?; Q7, When would you reduce or discontinue BZD following improvement in insomnia symptoms?; Q8, Which of the following factors would you consider as excusable reasons to continue a BZD?; Q9, Which strategy would you recommend for reducing or discontinuing BZD?; and Q10, Which medication would you recommend as an alternative if BZD is being reduced or discontinued? For each question, the available treatment choices were arranged according to the Japanese treatment guidelines for insomnia disorder and clinical practice. We invited certified psychiatrists from the Japanese Society of Clinical Neuropsychopharmacology and Japanese Society of Sleep Research, as well as councilors from the Japanese Society of Anxiety and Related Disorders throughout the country to participate in an email questionnaire survey from June 29, 2022 to July 31, 2022. The experts who agreed to participate were asked to evaluate each treatment choice using a nine-point Likert scale (1, “strongly not-recommended” to 9, “strongly recommended”). The clinical questions and treatment choices provided were presented in several tables. The survey took approximately 15 min to complete. The experts voluntarily participated in the survey with no incentives and were also asked to reveal their age, gender, and affiliated societies. This study was approved by the institutional review board of St. Luke’s International University (approval number: 2021-604).

### 2.2. Analysis

The following values were calculated for each treatment option: mean, standard deviation, 95% confidence interval (CI), and number of rating categories (i.e., not-recommended: responses 1–3; neutral: responses 4–6; and recommended: responses 7–9). Pearson’s chi-squared test was used to compare the numbers of these three rating categories for each treatment choice. When the responses were evenly distributed across the three categories with a *p*-value ≥ 0.05, “no consensus” was given for the corresponding clinical question, indicating a controversial strategy. Treatment options with 95% CI values ≥ 6.5 were regarded as “first-line recommendations,” indicating a consensus among experts for a particular situation. Options rated as 9 by > 50% of responders were defined as “treatments of choice,” indicating a particularly strong first-line recommendation. Options with 95% CI values ≥ 3.5 were regarded as “second-line treatments,” indicating reasonable options for patients who do not respond to or cannot tolerate first-line strategies. Treatment options with 95% CI values < 3.5 were considered “third-line treatments” (not-recommended) indicating that they are inappropriate options or used only when other options are ineffective.

## 3. Results

### 3.1. Participant characteristics

One hundred and ninety-five experts completed the survey. The mean age of respondents was 52.5 ± 9.5 years, and the proportions of men and women were 84.1 and 14.9%, respectively. Of the respondents, 89 (45.6%) were certified psychiatrists from the Japanese Society of Clinical Neuropsychopharmacology, 102 (52.3%) were certified psychiatrists from the Japanese Society of Sleep Research, and 13 (6.7%) were counselors from the Japanese Society of Anxiety and Related Disorders.

### 3.2. Primary treatment strategy for insomnia disorder

Regarding the primary pharmacological treatment for sleep initiation in insomnia, lemborexant (7.3 ± 2.0) was categorized as a first-line recommendation; eszopiclone (6.2 ± 1.8), suvorexant (6.0 ± 2.1), zopiclone (4.7 ± 2.0), and kampo were categorized as second-line treatments; and ramelteon (5.4 ± 2.2) and zolpidem (4.9 ± 2.2) were categorized as having “no consensus.” Other BZDs, including trazodone and quetiapine, were categorized as third-line treatments (not-recommended) (Table 1). Similarly, regarding the primary pharmacological treatment for sleep maintenance insomnia, lemborexant (7.3 ± 1.8) and suvorexant (6.8 ± 1.8) were categorized as first-line recommendations; eszopiclone (5.2 ± 2.0), quetiapine (4.0 ± 2.3), and kampo (3.9 ± 2.2) were categorized as second-line treatments; and ramelteon (5.2 ± 2.2) and trazodone (4.8 ± 2.3) were categorized as having “no consensus.” Other BZDs were categorized as third-line treatments (not-recommended) (Table 2). Moreover, recommendations for pharmacological treatment according to pharmacological categories are shown in the Supplementary Tables 1, 2.

**TABLE 1:** (Q1) Which pharmacological treatments would you recommend for sleep initiation insomnia in primary treatment?

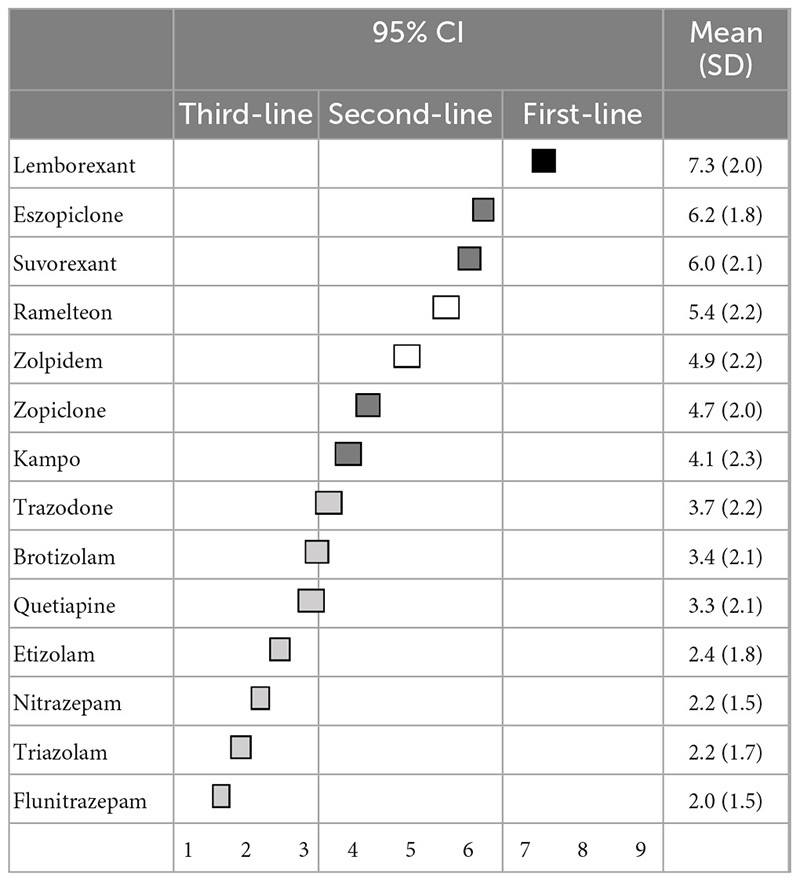

CI, confidence interval; SD, standard deviation.

**TABLE 2:** (Q2) Which pharmacological treatments would you recommend for sleep maintenance insomnia in primary treatment?

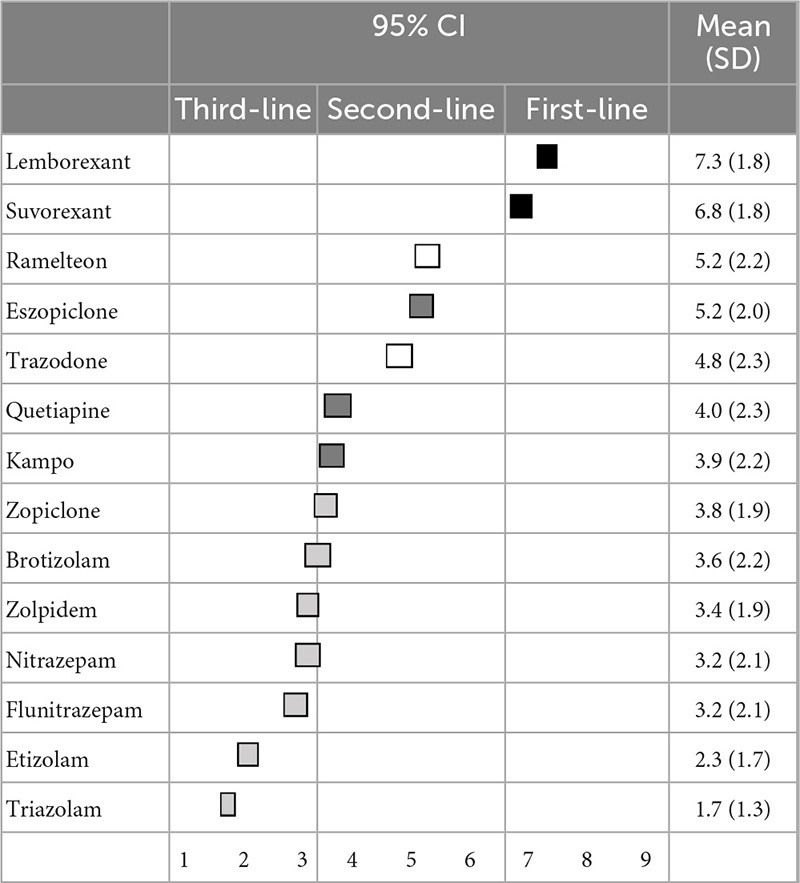

CI, confidence interval; SD, standard deviation.

Regarding non-pharmacological treatments for sleep initiation in insomnia, sleep hygiene education (8.4 ± 1.1) and relaxation therapy (7.0 ± 2.0) were categorized as first-line recommendations; stimulus control (6.5 ± 2.1), sleep restriction therapy (6.4 ± 2.2), and multicomponent CBT-I (5.6 ± 2.3) were categorized as second-line treatments; and sleep hygiene education was categorized as a “treatment of choice” (Table 3). Similarly, for sleep maintenance insomnia, sleep hygiene education (8.1 ± 1.5) was categorized as a first-line recommendation; and relaxation therapy (6.6 ± 2.1), sleep restriction therapy (6.5 ± 2.3), stimulus control (6.2 ± 2.2), and multicomponent CBT-I (5.7 ± 2.4) were categorized as second-line treatments (Table 4).

**TABLE 3:** (Q3) Which non-pharmacological treatments would you recommend for sleep initiating insomnia in primary treatment?

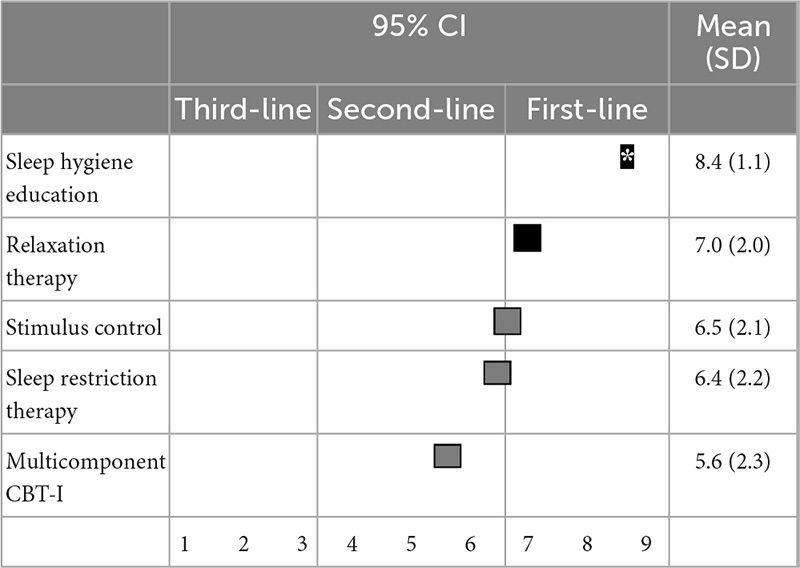

CBT-I, cognitive behavioral therapy for insomnia; CI, confidence interval; SD, standard deviation. *Treatment choice.

**TABLE 4:** (Q4) Which non-pharmacological treatments would you recommend for sleep maintenance insomnia in primary treatment?

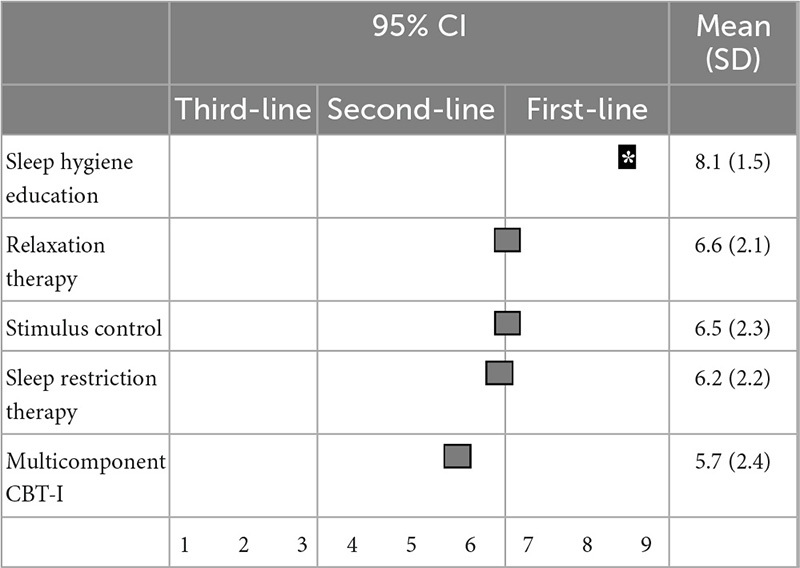

BT-I, cognitive behavioral therapy for insomnia; CI, confidence interval; SD, standard deviation.

*Treatment choice.

### 3.3. Treatment strategy when BZD hypnotics are ineffective

When BZD hypnotics do not improve the symptoms of insomnia, there was no first-line recommendation. Switching to lemborexant (6.7 ± 2.2) and suvorexant (6.1 ± 2.3), as well as combination treatment with lemborexant (6.3 ± 2.3) and suvorexant (5.9 ± 2.3), were categorized as second-line treatments. Additionally, increasing the dose of BZD hypnotics was also categorized as second-line treatment. Switching to other medications, trazodone (5.3 ± 2.4), other BZDs (4.9 ± 2.4), quetiapine (4.7 ± 2.5), and ramelteon (4.5 ± 2.3) were categorized as having “no consensus.” Combination treatment with trazodone (5.4 ± 2.5), ramelteon (5.1 ± 2.4), and quetiapine (4.7 ± 2.5) were also categorized as having “no consensus.” Only combination treatment with other BZDs (3.0 ± 2.2) was categorized as a third-line treatment (not-recommended) (Table 5).

**TABLE 5:** (Q5) Which pharmacological treatments would you recommend if BZDs do not improve insomnia symptoms?

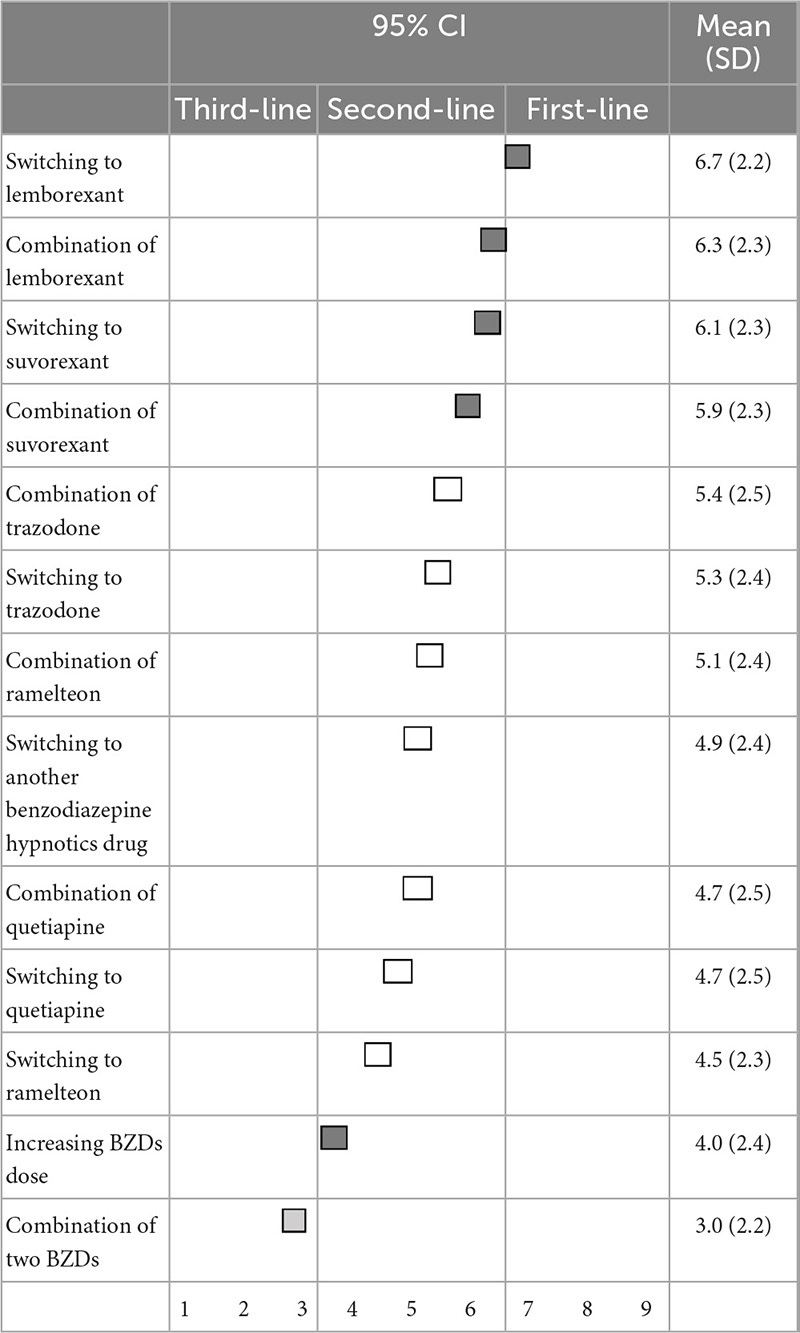

BZDs, Benzodiazepines and benzodiazepine receptor agonists; CI, confidence interval; SD, standard deviation.

For non-pharmacological treatment, the differential diagnosis of other psychiatric disorders (8.2 ± 1.3), sleep hygiene education (8.1 ± 1.4), differential diagnosis of other sleep disorders (8.0 ± 1.6), and relaxation therapy (7.0 ± 1.9) were categorized as first-line recommendations. Consultation with a specialist (6.8 ± 2.0), sleep restriction therapy (6.5 ± 2.2), stimulus control (6.5 ± 2.1), and multicomponent CBT-I (5.9 ± 2.4) were categorized as second-line treatments. Differential diagnosis of other psychiatric disorders, sleep hygiene education, and differential diagnosis of other sleep disorders were all categorized as “treatments of choice” (Table 6).

**TABLE 6:** (Q6) Which non-pharmacological treatments would you recommend if BZDs do not improve insomnia symptoms?

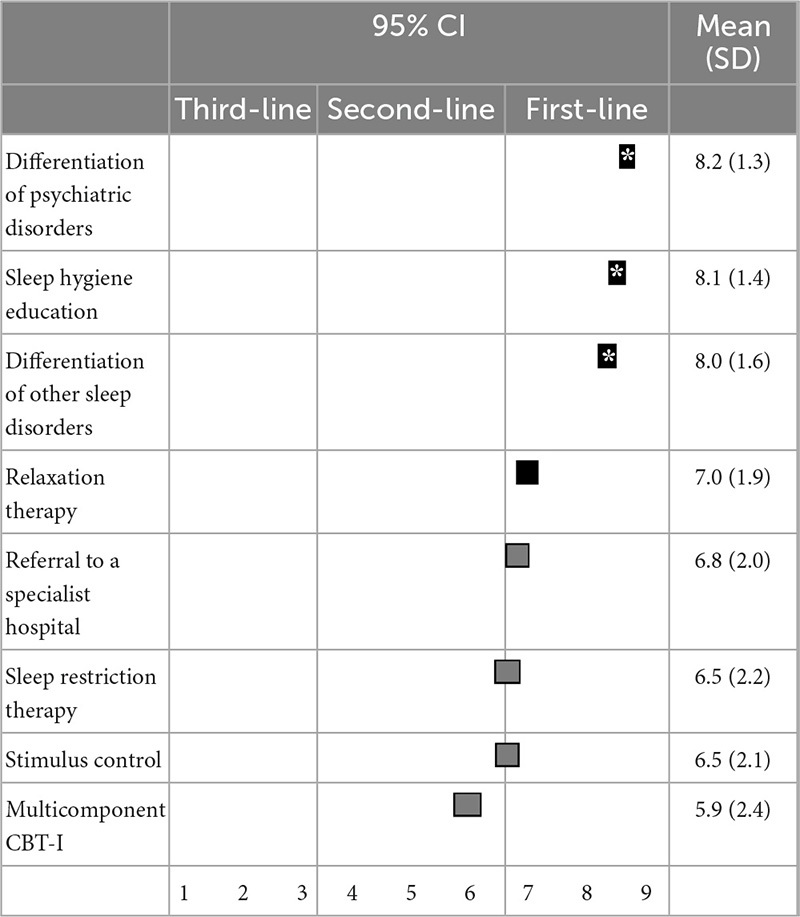

BZDs, Benzodiazepines and benzodiazepine receptor agonists; CBT-I, cognitive behavioral therapy for insomnia; CI, confidence interval; SD, standard deviation.

*Treatment choice.

### 3.4. Discontinuation of BZD hypnotics

There was no first-line recommendation for the timing of reducing or discontinuation of BZD hypnotics following improvement in insomnia symptoms. As for timing, 1–3 months (6.4 ± 2.0), 3–6 months (5.9 ± 2.1), and immediately (4.2 ± 2.3) after improvement in symptoms were categorized as second-line treatments, while 6–12 months (5.1 ± 2.4) after improvements in symptoms were categorized as having “no consensus.” Only a timing of >1 year after improvement in symptoms (3.5 ± 2.2) was categorized as a third-line treatment (not-recommended) (Table 7).

**TABLE 7:** (Q7) When would you reduce or discontinue BZDs after insomnia symptoms improve?

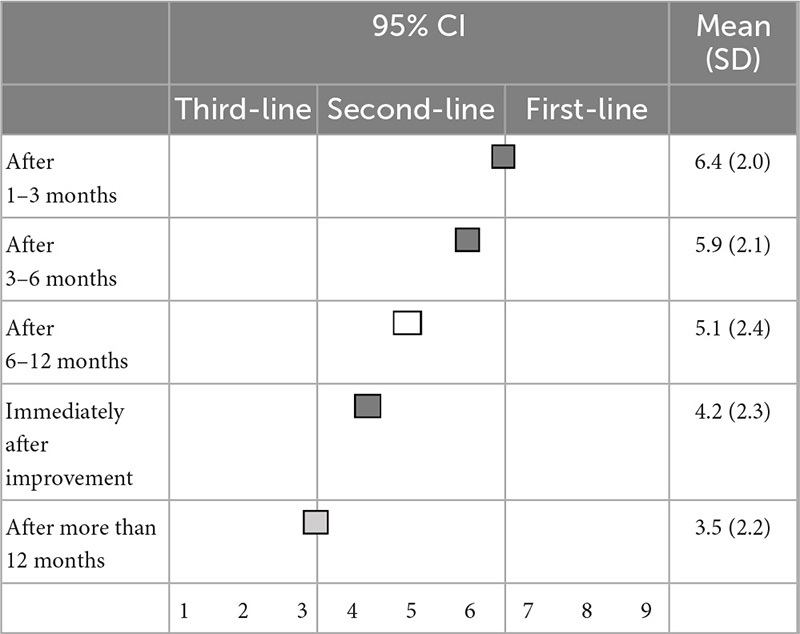

BZDs, Benzodiazepines and benzodiazepine receptor agonists; CI, confidence interval; SD, standard deviation.

Regarding the suggested excusable reasons for continuing BZD hypnotics, anticipation of physical or mental deterioration upon discontinuation (6.9 ± 1.8) was categorized as the first-line recommendation; and a history of insomnia symptom relapse when discontinuing hypnotics (6.4 ± 1.9), unstable physical or mental states or quality of life (6.1 ± 2.1), monotherapy or low dose use of hypnotics (5.8 ± 2.0), desire to continue hypnotics (5.2 ± 2.1), and no serious side effects (4.9 ± 2.1) were categorized as second-line treatments (Table 8).

**TABLE 8:** (Q8) Which of the following factors would you consider as excusable reasons to continue BZDs?

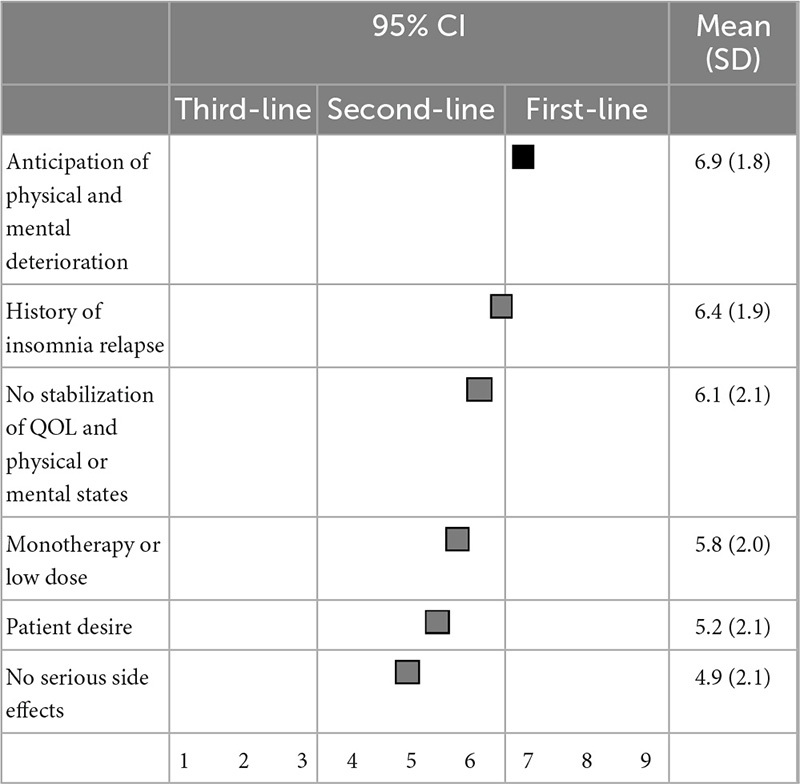

BZDs, Benzodiazepines and benzodiazepine receptor agonists; CI, confidence interval; QOL, quality of life; SD, standard deviation.

When reducing or discontinuing BZD hypnotics, gradual reduction (8.1 ± 1.2) and sleep hygiene education (7.9 ± 1.5) were categorized as first-line recommendations. Relaxation therapy, switching to other hypnotics, sleep restriction therapy, switching to as-needed use of hypnotics, stimulus control, and patients’ self-adjustment of hypnotics were categorized as second-line treatments (Table 9).

**TABLE 9:** (Q9) Which strategy would you recommend for reducing or discontinuing a BZD?

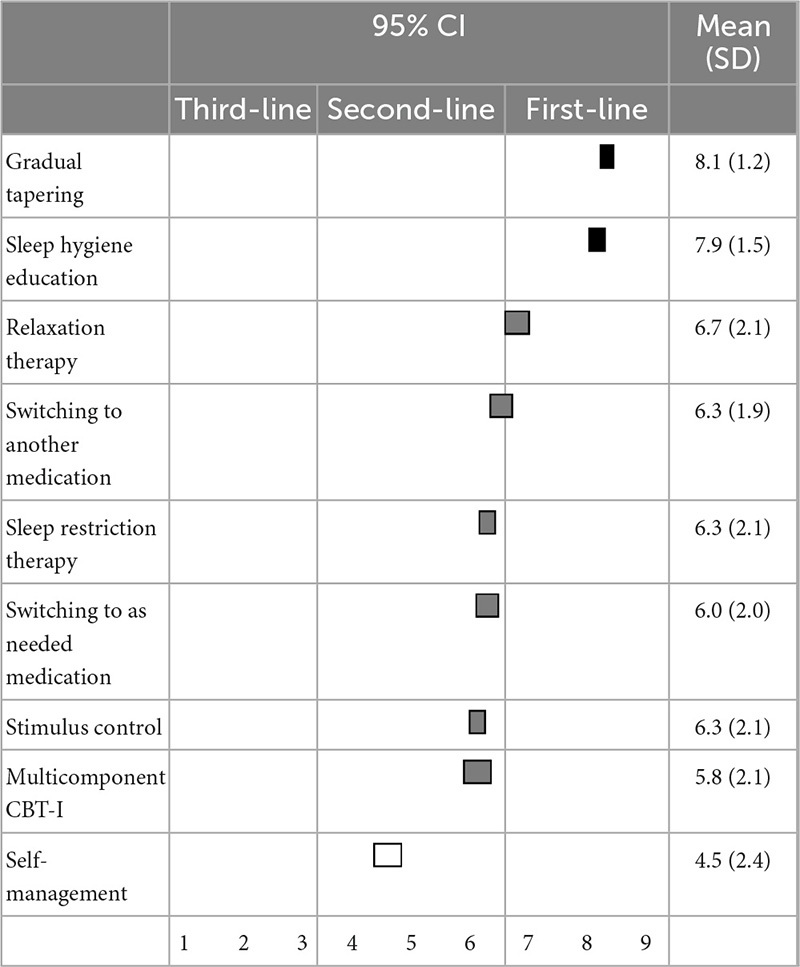

BZDs, Benzodiazepines and benzodiazepine receptor agonists; CBT-I, cognitive behavioral therapy for insomnia; CI, confidence interval; SD, standard deviation.

When reducing or discontinuing BZD hypnotics while switching to other medications, lemborexant (7.5 ± 1.8) and suvorexant (6.9 ± 1.9) were categorized as first-line recommendations. Switching to ramelteon (5.7 ± 2.3) was categorized as a second-line treatment, while trazodone (5.3 ± 2.3), quetiapine (4.4 ± 2.4), and kampo (4.4 ± 2.5) categorized as having “no consensus” (Table 10).

**TABLE 10:** (Q10) Which medication would you recommend for switching if BZDs are reduced or discontinued?

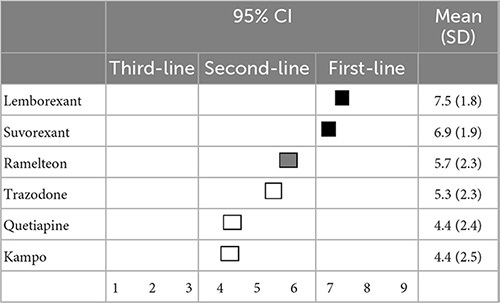

BZDs, Benzodiazepines and benzodiazepine receptor agonists; CI, confidence interval; SD, standard deviation.

## 4. Discussion

This is the first study to show recommendations by expert consensus for both pharmacological and non-pharmacological treatments for insomnia disorder depending on individual clinical situations. Lemborexant is the first-line recommendation for the primary treatment of both sleep initiation and sleep maintenance insomnia. Suvorexant is a first-line recommendation of primary treatment for sleep maintenance insomnia. When reducing or discontinuing BZD hypnotics while switching to other medications, lemborexant and suvorexant were the first-line recommendations. Regarding non-pharmacological treatment, sleep hygiene education is a first-line recommendation for both sleep initiation and sleep maintenance insomnia. When reducing or discontinuing BZD hypnotics, a gradual reduction and sleep hygiene education were the first-line recommendations. Although the recommendations were not based on scientific evidence, it could be easy for clinicians to interpret and implement these results in various real-world settings.

For primary pharmacological treatment, orexin receptor antagonists were the first-line treatment. Although it is difficult to compare the usefulness of lemborexant and that suvorexant, the experts in our study were likely to consider that lemborexant was more useful than suvorexant. Moreover, eszopiclone, a BZD receptor agonist, was useful both in sleep initiation and sleep maintenance insomnia, despite most other BZDs being third-line treatments (not-recommended) or having “no consensus.” Notably, a recent network meta-analysis that compared the acute and long-term effectiveness of 36 pharmacological treatments for insomnia disorder suggested that eszopiclone and lemborexant had a favorable profile, but eszopiclone may cause substantial adverse events, and the safety data on lemborexant were inconclusive (17), which is in line with our results that showed the usefulness of lemborexant and eszopiclone. Thus, the results of our study, which are based on expert opinion, could be linked to evidence-based recommendations.

Regarding primary non-pharmacological treatment, sleep hygiene education was the first-line treatment for both sleep initiation and sleep maintenance insomnia. Multicomponent CBT-I was the lowest recommendation of all non-pharmacological treatments, although CBT-I was classified as a second-line treatment. The American Academy of Sleep Medicine guidelines on psychological and behavioral treatments suggest that multicomponent CBT-I is strongly recommended and that stimulus control, sleep restriction therapy, and relaxation therapy—which are part of CBT-I—are conditionally recommended for the treatment of insomnia disorder, while sleep hygiene alone is not recommended for the treatment of insomnia disorder (15). Although the reason behind this discrepancy is unclear, it is possible that there is an evidence-practice gap between the recommendation of the American Academy of Sleep Medicine and that of the Japanese expert consensus. Although Japanese experts understand the effectiveness of multicomponent CBT-I based on scientific evidence, it is difficult for them to strongly recommend multicomponent CBT-I because of the lack of resources available for multicomponent CBT-I in Japanese clinical settings.

Although BZDs, especially zolpidem and eszopiclone, were recommended in clinical guidelines in the short-term because of the relatively good risk-to-benefit ratio (2), the long-term use of these medications was not recommended due to concerns pertaining to adverse events, including dependence or tolerance. Their use was restricted to within 4 weeks in many countries (18). In contrast, there was no restriction for the long-term prescription of BZDs in Japan, even though most specialists for insomnia were against the long-term use of these medications. Considering these situations in Japan, this consensus began with the predetermined recommendation that a treatment strategy to avoid the use of BZD hypnotics and implement alternative treatment to discontinue BZD hypnotics is necessary for the proper management of insomnia disorder in clinical settings. The results of this study show that most specialists recommend BZDs to be discontinued within 6 months after improving insomnia symptoms. However, Japanese specialists also suggest that it was difficult and unreasonable to discontinue all long-term use of BZDs from patients, especially those who were anticipated to have worsened physical and mental condition if withdrawal occurred.

When reducing or discontinuing BZDs, the experts also considered sleep hygiene education and gradual tapering as first-line recommendations, while stimulus control, sleep restriction therapy, relaxation therapy, and multicomponent CBT-I were second-line treatments. Our previous systematic review and meta-analysis suggested that CBT-I plus gradual tapering was more effective for discontinuing BZD hypnotics than was sleep hygiene plus gradual tapering (risk ratio: 1.68; 95% confidence interval: 1.19–2.39, *p* = 0.003) (19). This discrepancy could also be caused by an evidence-practice gap, especially in primary care settings, due to the lack of resources in Japan. Therefore, Japanese experts may suggest that primary care physicians should first try discontinuing BZDs using sleep hygiene education plus gradual tapering. Accordingly, if patient have difficulty withdrawing from BZD hypnotics, they can be referred to a specialist for CBT-I.

When reducing or discontinuing BZD hypnotics while switching to other medications, orexin receptor antagonists were the first-line recommendation. Some limited studies with low evidence levels have reported that suvorexant (20) and lemborexant (21) might be effective for discontinuing BZDs, though it is difficult to draw conclusions about the effectiveness of orexin receptor antagonists for discontinuing BZDs. Further studies are necessary to confirm the effectiveness of orexin receptor antagonists for discontinuing BZDs.

Our study has several limitations. First, expert consensus for the management of insomnia disorder could be ranked as a low level of evidence in clinical guidelines. Second, a lack of adequate information in some clinical questions may have affected the ability of respondents to choose proper treatment. Patient heterogeneity should be considered when the recommendations of this expert consensus are applied. Third, because all participating experts were involved in Japanese medical care, it is difficult to generalize this result for the global population of patients with insomnia disorder. Finally, the analytical methods and rating categories [i.e., 1–3 (disagree), 4–6 (neutral), and 7–9 (agree)] used in this study were somewhat arbitrary. Last, although some of the 196 experts who answered the questionnaire had potential conflict of interests, which may have affected the results of this expert consensus, we did not query about potential conflicts of interest among all 196 experts.

## 5. Conclusion

Japanese experts recommend lemborexant for both sleep initiation and sleep maintenance insomnia at the start of treatment and when discontinuing BZDs. Suvorexant is recommended for sleep maintenance insomnia when starting the treatment and when discontinuing BZDs. Sleep hygiene education is strongly recommended in all situations. CBT-I is conditionally recommended because of the lack of resources for the initialization of treatment, for treatment-resistant insomnia, and when discontinuing BZDs. This information could help physicians who feel uncertain when deciding on treatment plans in clinical settings to improve patient outcomes.

## Data availability statement

The raw data supporting the conclusions of this article will be made available by the authors, without undue reservation.

## Ethics statement

The studies involving human participants were reviewed and approved by the Institutional Review Board of St. Luke’s International University. The patients/participants provided their written informed consent to participate in this study.

## Author contributions

YT drafted the manuscript, organized and implementation of this study, and interpreted the data. YA, KIe, KIn, ES, KMi, and KW collected the data. MT, KMa, TU, AS, IO, NK, HY, MS, and KK interpreted the data. YT and HS performed the statistical analysis. All authors took part in revising the manuscript and approved the final version of the manuscript.
