# Supplementary material for: Treatment strategy for insomnia disorder: Japanese expert consensus
Source: Front Psychiatry. 2023 May 9;14:1168100. doi: 10.3389/fpsyt.2023.1168100 (PMC10203548; doi:10.3389/fpsyt.2023.1168100)
Supplement: Supplementary file 1 [file Table_1.DOCX]

|  | 95% CI | | | Mean (SD) |
| --- | --- | --- | --- | --- |
|  | Third-line | Second-line | First-line |  |
| Orexin receptor antagonists |  | | | 6.7(1.8) |
| Melatonin receptor agonists |  | | | 5.4(2.2) |
| Nonbenzodiazepine |  | | | 5.3(1.7) |
| Kanpo |  | | | 4.1(2.3) |
| Antidepressant |  | | | 3.7(2.2) |
| Antipsychotic drugs |  | | | 3.3(2.1) |
| Benzodiazepine | ^1 2 3 4 5 6 7 8 9^ | | | 2.4(1.4) |

Supplementary table S1 (Q1) Which pharmacological treatments would you recommend for sleep initiation insomnia in primary treatment?

CI, confidence interval; SD, standard deviation

Supplementary table S2 (Q2) Which pharmacological treatments would you recommend for sleep initiation maintenance insomnia in primary treatment?

|  | 95% CI | | | Mean (SD) |
| --- | --- | --- | --- | --- |
|  | Third-line | Second-line | First-line |  |
| Orexin receptor antagonists |  | | | 7.1(1.7) |
| Melatonin receptor agonists |  | | | 5.2(2.2) |
| Antidepressant |  | | | 4.8(2.3) |
| Nonbenzodiazepine |  | | | 4.1(1.6) |
| Antipsychotic drugs |  | | | 4.0(2.3) |
| Kanpo |  | | | 3.9(2.2) |
| Benzodiazepine | ^1 2 3 4 5 6 7 8 9^ | | | 2.8(1.5) |

CI, confidence interval; SD, standard deviation
